# Examining the impact of the COVID-19 pandemic on hospital-associated *Clostridioides difficile* infection

**DOI:** 10.1017/ice.2024.128

**Published:** 2024-12

**Authors:** Michael J. Ray, Jon P. Furuno, Luke C. Strnad, Eric T. Lofgren, Jessina C. McGregor

**Affiliations:** 1 Oregon State University College of Pharmacy, Department of Pharmacy Practice, Portland, OR, USA; 2 Oregon Health & Science University-Portland State University School of Public Health, Portland, OR, USA; 3 Oregon Health & Science University School of Medicine, Division of Infectious Diseases, Portland, OR, USA; 4 Washington State University Allen School for Global Health, Pullman, WA, USA

## Abstract

**Objective:**

To evaluate the impact of changes in the size and characteristics of the hospitalized patient population during the COVID-19 pandemic on the incidence of hospital-associated *Clostridioides difficile* infection (HA-CDI).

**Design:**

Interrupted time-series analysis.

**Setting:**

A 576-bed academic medical center in Portland, Oregon.

**Methods:**

We established March 23, 2020 as our pandemic onset and included 24 pre-pandemic and 24 pandemic-era 30-day intervals. We built an autoregressive segmented regression model to evaluate immediate and gradual changes in HA-CDI rate during the pandemic while controlling for changes in known CDI risk factors.

**Results:**

We observed 4.5 HA-CDI cases per 10,000 patient-days in the two years prior to the pandemic and 4.7 cases per 10,000 patient-days in the first two years of the pandemic. According to our adjusted segmented regression model, there were neither significant changes in HA-CDI rate at the onset of the pandemic (level-change coefficient = 0.70, *P*-value = 0.57) nor overtime during the pandemic (slope-change coefficient = 0.003, *P*-value = 0.97). We observed significant increases in frequency and intensity of antibiotic use, time at risk, comorbidities, and patient age before and after the pandemic onset. Frequency of *C. difficile* testing did not significantly change during the pandemic (*P*= 0.72).

**Conclusions:**

Despite large increases in several CDI risk factors, we did not observe the expected corresponding changes in HA-CDI rate during the first two years of the COVID-19 pandemic. We hypothesize that infection prevention measures responding to COVID-19 played a role in CDI prevention.

## Introduction

In the hospital setting, *Clostridioides difficile* is challenging to eliminate from the environment, making hand hygiene, personal protective equipment (PPE), and environmental cleaning crucial components to mitigating hospital-associated *C. difficile* infection (HA-CDI).^1^ However, the primary patient-level modifiable risk factor for HA-CDI is antibiotic use, as exposure to broad-spectrum antibiotic therapy can increase the risk of CDI up to four-fold.^2^ Thus, HA-CDI is a frequently measured outcome in evaluations of interventions aimed at optimizing inpatient antibiotic use.^1–4^

Though HA-CDI has been extensively studied, the COVID-19 pandemic significantly altered the healthcare context. In the United States, April–June 2020 saw a 150 percent decrease in hospital admissions, and a 40 percent increase in ICU admissions. With this came nearly a 50 percent increase in all-cause in-hospital deaths, a third of which were COVID-19-related.^5,6^ Frequently reported were staffing shortages, repurposing of nonICU beds, and overall substandard care for non-COVID-19 patients relative to COVID-19 patients.^7^ This resulted in a shift in the hospitalized patient case-mix that directly influenced both modifiable and non-modifiable risk factors providing an opportunity to study the impact of these changes on the risk of CDI. This includes fluctuations in the frequency of patients with advanced age, immunosuppression, use of acid-suppressing medication, and comorbid conditions.^8^ Understanding this changing context can provide new insight into the complex epidemiology of HA-CDI.

Our study objectives were to describe changes in HA-CDI rate after healthcare changes due to the COVID-19 pandemic and to determine the relative importance of the contextual factors likely associated with CDI. We hypothesized that we would see an increase in HA-CDI rate at the onset of the pandemic due to increases in antibiotic use, followed by a gradual return to the baseline rate.

## Methods

### Setting and study design

We conducted an interrupted time-series analysis using retrospective healthcare data from Oregon Health & Science University (OHSU) Hospital, a 576-bed academic hospital in Portland, OR. We collected medical record data from our institution’s research data repository for inpatient visits between January 2018 and April 2022. We limited our analysis to adult (≥ 18 years) inpatients and excluded patients with hospital stays under 4 days and those known to have recurrent (CDI in the previous 8 weeks) or community-acquired (CDI diagnosis within first 3 d of hospitalization) CDI. We aggregated data into 30-day periods to ensure we were evaluating uniform time intervals. This project was approved by OHSU’s Institutional Review Board (OHSU IRB #23278).

### Interruption timepoint

We established March 23, 2020 as our primary interruption timepoint (hereafter referred to as “pandemic onset”), the date the Oregon Governor issued an executive order prohibiting elective and non-urgent procedures as well as non-essential visitation.^9^ We established the 24 30-day intervals prior to the interruption point as our “pre-pandemic” period, beginning March 4, 2018, and an equal number of 30-day intervals after the interruption point as our post-interruption, “pandemic era” period ending March 13, 2022. We also evaluated the following dates as secondary pandemic interruption points: vaccine rollout (December 18, 2020), “pandemic year 2” (March 23, 2021), delta wave (June 1, 2021), and omicron wave (December 20, 2021).^10^

Our outcome was incident, non-recurrent HA-CDI per 10,000 patient-days for each time period. We identified incident, non-recurrent cases of HA-CDI using a previously validated combination of medication and laboratory testing data.^11^ Incident CDI cases were hospital-associated if the onset date, defined as the date of first anti-*C. difficile* antibiotic administration or stool specimen sample collection from the positive *C. difficile* laboratory test, whichever occurred first, occurred on hospital day 4 or later, consistent with the National Healthcare Safety Network (NHSN) definition.^12^ We considered cases non-recurrent if no prior CDI events were identified at the index facility in the 8 weeks before the initial CDI diagnosis. We aggregated HA-CDI counts into 30-day intervals and divided by number of patient days to get our primary outcome variable.

### Antibiotic prescribing

To describe intensity of antibiotic therapy, we utilized the antibiotic spectrum index (ASI) developed by Gerber et al.^13^ ASI assigns and sums point values (ranging from 1 to 13) based on each agent’s activity against a variety of bacterial species. Higher ASI values represent broader spectrum antibiotics. We calculated each hospitalized patient’s ASI per antibiotic day by summing ASI scores for each individual agent a patient was exposed to across all days of therapy and then dividing by the number of hospital days during which a patient received at least one antibiotic, consistent with how the variable was manipulated in the original article.^13^

### Other CDI risk factors

We examined other known CDI risk factors including time at-risk,^14^ age^8,15^ number of comorbid conditions (defined by Elixhauser comorbidity index^16^),^15,17^ days hospitalized in the previous 8 weeks,^18,19^ inpatient antibiotic use in the previous 8 weeks;^20^ proton pump inhibitor or H2-receptor antagonist use,^15,17,21^ nasogastric tube placement,^1,15,21^ corticosteroid use,^17^ or chemotherapy (yes or no);^8^ source of hospital admission (Emergency Department, other healthcare facility, non-healthcare),^22,23^ and *C. difficile* colonization pressure (total case-days).^24,25^ We defined colonization pressure as the total daily number of patients with CDI present in the same ward during each patient’s time at risk. A patient with CDI or colonized with *C. difficile* was eligible to contribute to colonization pressure for the 14 days after initiation of first CDI treatment or until discharge. We summed the daily number of patients with CDI by hospital ward for every day a patient was present on the ward (*case-days* of colonization pressure,^25^ and aggregated all variables into 30-day intervals.

### Statistical analysis

We performed segmented autoregressive linear regression to examine the pre-interruption trend in HA-CDI rate, as well as post-interruption *slope/trend* change and *level* change using SAS v9.4 (Cary, NC). An autoregressive model accounts for the autocorrelated error terms that are inherent to time series data.^26^ We examined model parameter estimates and *P*-values to assess the evidence of slope and/or level changes in HA-CDI rate after the pandemic’s onset.

To assess changes in known HA-CDI risk factors, we generated identical segmented regression models with each risk factor as the dependent variable using the same parameters described above for the HA-CDI model. Any CDI risk factors with slope or level-change *P*-values greater than 0.15 were considered for inclusion in a multivariable model describing changes in HA-CDI rate over time. We then fit a multivariable model using the Akaike Information Criterion and total R^2^ values to assess model fit, minimizing the former and maximizing the latter. Variables for pre-pandemic trend, the pre- post-indicator variable, and pandemic-era trend were always included in the model.

Finally, we examined the frequency of *C. difficile* laboratory testing on a per encounter basis to ensure that testing did not change at pandemic’s onset, which could introduce detection bias. We defined a single encounter as an individual patient’s entire hospital stay. We also examined test positivity to identify any significant *C. difficile* trends not captured by our HA-CDI case definition.

## Results

We identified 254 cases of HA-CDI over the entire study period, corresponding to a rate of 4.2 cases per 10,000 patient-days (standard deviation (SD) = 2.2). In the pre-pandemic period, there were 137 HA-CDI cases identified, or 4.5 cases per 10,000 patient-days (SD = 1.8). There were 81 cases in the pandemic era, or 4.7 per 10,000 patient-days (SD = 3.2). Table 1 outlines patient characteristics for the two time periods. There was a significant decrease in the mean number of admissions per 30-day period with the onset of the pandemic (1441 vs 894) (Table 2, Appendix Figure 1). The pandemic-era patient population was, on average, older than the pre-pandemic population (57.8 vs 56.5 years) and had slightly longer average lengths of stay (9.2 d vs 8.8 d). We also observed a decrease in average case-days of colonization pressure per period (3.9 vs 1.1).

**Table 1.** Patient attributes before (March 4, 2018 to March 23, 2020) and during the first two years (March 24, 2020 to March 13, 2022) of the COVID-19 pandemic

Patient characteristic	Pre-pandemic	Pandemic era
Female sex (%)	52.7	53.3
White race (%)	87.1	85.9
Hispanic ethnicity (%)	7.4	7.7
Age (mean)	56.5	57.8
Number of comorbidities (mean)	1.96	2.01
Inpatient antibiotic use (%)	68.3	67.8
Transferred from ED (%)	24.1	16.8
Inpatient PPI or H2RA (%)	5.7	5.9
Chemotherapy procedures (%)	5.5	4.3
Nasogastric tube placement (%)	8.8	8.6
Any gastrointestinal procedure (%)	4.1	3.4

Abbreviations: ED, emergency department; PPI, proton pump inhibitor; H2RA, H2-histamine receptor antagonist.

**Table 2.** Pre-COVID-19 pandemic (March 4, 2018 to March 23, 2020) and pandemic-era (March 24, 2020 to March 13, 2022) means, slope, and level changes for HA-CDI and key risk factors

	Pre-period mean	Pandemic-era mean	Immediate pandemic-era change (95% confidence interval)	Pandemic-era trend change (95% confidence interval)
ASI points per antibiotic day	4.4	4.84	0.46 (0.33, 0.58)	−0.01 (−0.02, −0.003)
Number of antibiotic days per encounter	3.96	4.41	0.24 (0.01, 0.47)	0.02 (0.001, 0.034)
Days of therapy per encounter	5.78	7.24	1.12 (0.70, 1.54)	0.03 (−0.004, 0.05)
Admissions per month	1441	894	−444 (−585, -303)	−0.46 (−16.8, 15.9)
Time at-risk (days)	8.76	9.19	−0.02 (−0.42, 0.37)	0.06 (0.03, 0.09)
Number of comorbidities	1.96	2.01	0.11 (0.04, 0.18)	-0.010 (−0.01, −0.004)
Patient age (years) at admission	56.5	57.8	0.81 (−0.21, 1.83)	−0.09 (−0.18, 0.00)
Total case-days of colonization pressure	3.91	1.09	−1.03 (−2.171, 0.12)	0.05 (−0.04, 0.14)
HA-CDI rate (per 10,000 patient-days)	4.52	4.06	−0.88 (−3.84, 1.27)	0.09 (−0.11, 0.26)
*C. difficile* tests (count)	74.5	50.6	−15.62 (−25.3, −5.9)	0.03 (−0.67, 0.72)
*C. difficile* tests (per 100 admitted patients)	5.18	5.65	0.82 (0.06, 2.57)	−0.01 (−0.06, 0.04)
*C. difficile* test positivity (percent)	7.58	6.67	−2.85 (−0.23, 0.11)	0.27 (0.03, 0.50)

Abbreviations: ASI, antibiotic spectrum index; HA-CDI, hospital-associated *Clostridioides difficile* infection.

### Changes in HA-CDI risk factors

**Figure 1.**
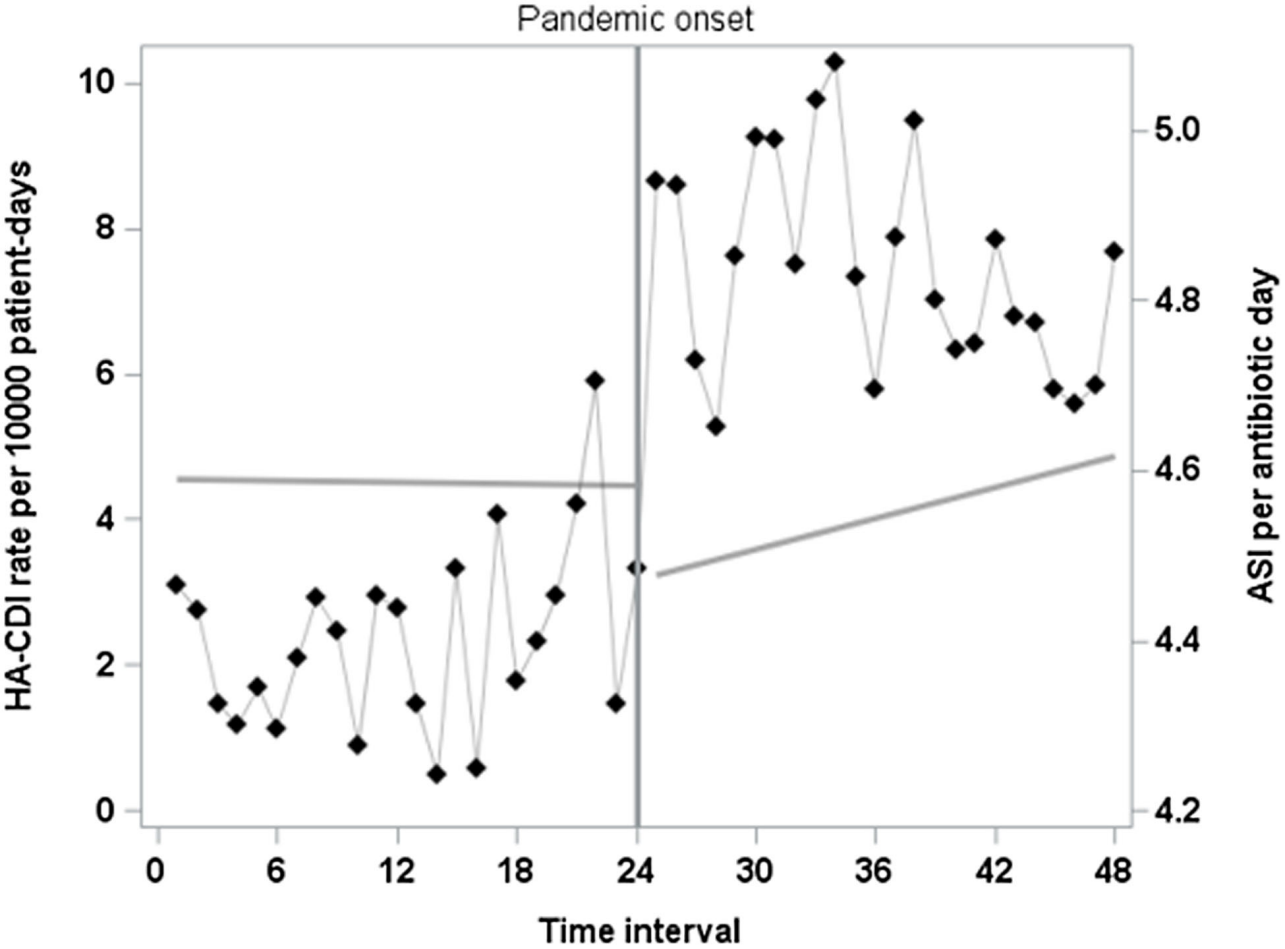
Time series overlay of HA-CDI rate antibiotic spectrum index points per antibiotic day before and during the pandemic.

According to our unadjusted autoregressive segmented regression model, there were neither significant immediate changes in HA-CDI rate at the onset of the pandemic (level-change coefficient = -0.88, *P* = 0.36), nor were there significant changes in pandemic-era slope over time (slope-change coefficient = 0.09, *P* = 0.18). We did observe significant changes in several CDI risk factors (Table 2), including intensity and frequency of antibiotic therapy (Figure 1). This includes a level increase in ASI per antibiotic day (0.46 ASI points per antibiotic day, Table 2), and a slope (0.02 additional antibiotic days per encounter per interval) and level (0.24 additional antibiotic days per encounter) increase in number of antibiotic days per encounter (Table 2).

We observed an increase in the mean number of comorbid conditions (0.11 additional comorbidities, on average, pre-pandemic mean comorbid condition range: 1.8 – 2.0, pandemic-era range: 1.9 – 2.2) at the pandemic’s onset followed by a gradual return to the pre-pandemic mean (Appendix Figure 2). We did not observe immediate level changes in mean time at-risk, but we did observe a significant pandemic-era slope increase (0.06 additional days per interval). We observed a small level decrease in case-days of colonization pressure throughout our entire study period (1.03 fewer case-days), though this could be due to chance (*P* = 0.09) (Table 2).

### Multivariable segmented regression results

The multivariable model included average case-days of colonization pressure per 30-day period, average ASI per antibiotic day, and average number of comorbid conditions (Figure 1). After adjusting for colonization pressure, ASI per antibiotic day, and mean number of comorbidities, there was no immediate change in HA-CDI at the pandemic’s onset (level-change coefficient = 0.70, *P*-value = 0.57) nor was there a change in slope (slope-change coefficient = 0.003, *P*-value = 0.97).

### C. difficile testing

We evaluated the frequency of *C. difficile* testing over time to assess the potential for detection bias. While there was an initial drop in the total volume of *C. difficile* testing at the onset of the pandemic, (level-change coefficient = -15.6, *P*-value = 0.003), mean testing frequency increased on a per-encounter basis (pre/post mean 5.2 vs 5.7, level-change coefficient = 0.82, *P*-value = 0.04).

### Evaluation of additional interruption time points

**Figure 2.**
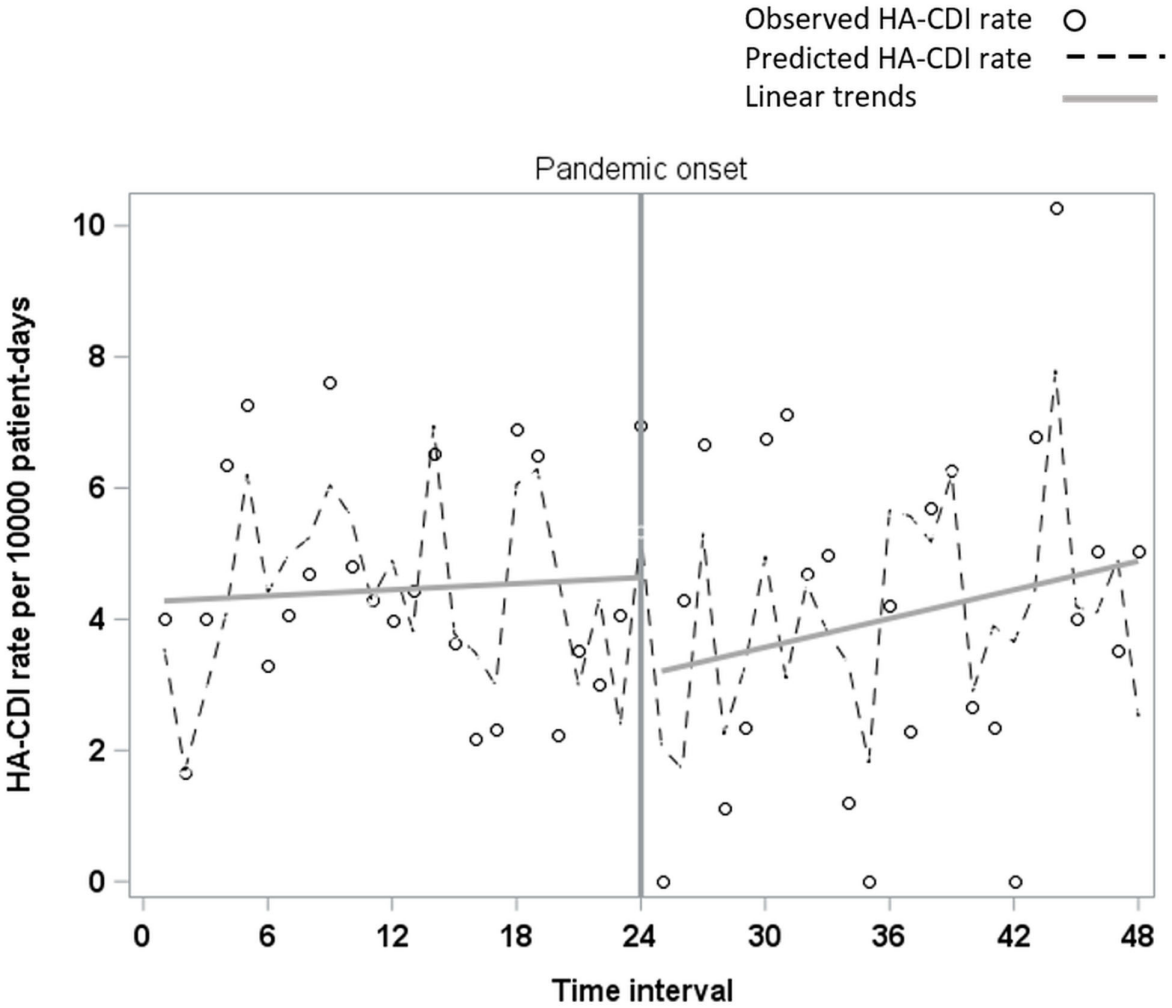
Time series of HA-CDI rate before and throughout the first two years of the COVID-19 pandemic with trendlines utilizing final adjusted model. R^2^ = 0.47; adjusted for antibiotic spectrum index points per antibiotic day, case-days of colonization pressure, and sum of comorbidities.

**Figure 3.**
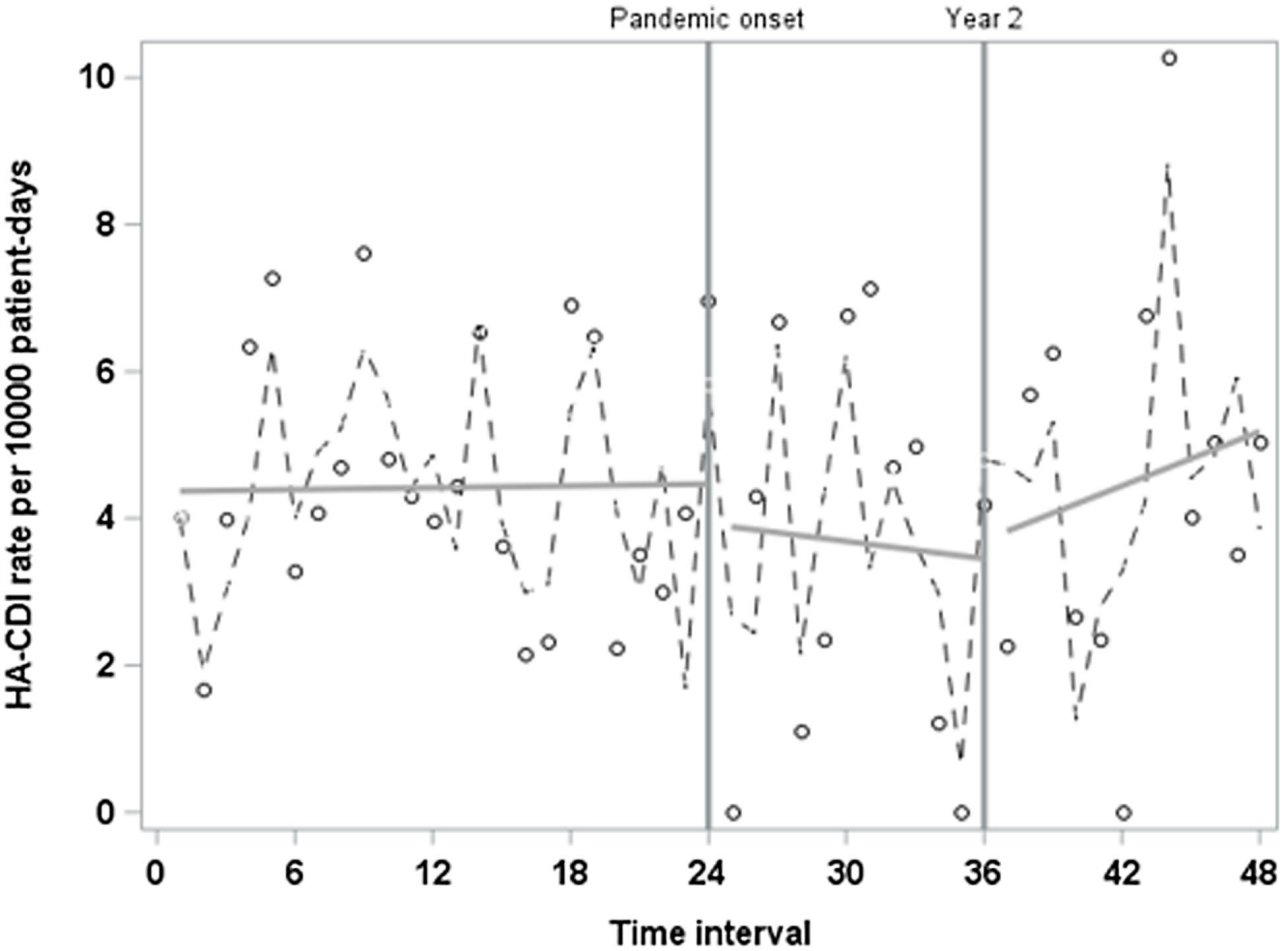
Results of segmented regression model with an additional interruption point at pandemic year 2. R^2^ = 0.56; p-value for year 2 trend change = 0.005.

Inspection of our time series data suggested an increase in HA-CDI trend approximately one year into the pandemic (Figure 2). Thus, we evaluated an additional interruption point at the start of pandemic year 2 within the final regression model. We observed a significant slope increase in our final 12 time intervals (slope-change coefficient = 0.36, *P* = 0.005). Adding terms for the second pandemic-era period also explained more variability in our time series data (R-squared 0.56 vs 0.47) (Figure 3). None of the other secondary interruption points evaluated yielded significant results.

## Discussion

Despite increases in CDI risk factors at the onset of the COVID-19 pandemic, we did not observe significant changes in HA-CDI rate controlling for intensity of antibiotic prescribing, colonization pressure, and number of comorbidities. The increases in key HA-CDI risk factors included frequency and intensity of antibiotic use, patient comorbidity burden, and time at risk. We also saw a slight level decrease in case-days of *C. difficile* colonization pressure at the pandemic’s onset, though the observed change could be due to chance.

The increase in ASI per antibiotic day is noteworthy. Though the proportion of patients receiving any inpatient antibiotic did not change, antibiotics were administered on more calendar days during the pandemic era compared to pre-pandemic at our institution. Thus, the only way for this variable to increase would be the prescribing of broader spectrum agents or a greater number of separate agents on the same calendar day. This can be interpreted as an increase in the intensity of antibiotic therapy throughout the pandemic. While we would expect a corresponding increase in HA-CDI, we did not observe this. Current literature on antibiotic use during the pandemic shows vast heterogeneity. There were documented shifts in antibiotic utilization for suspected COVID-19 cases. Overall antibiotic use in hospitals increased early in the pandemic, with a 5 percent increase in overall antibiotic prescribing and a 22 percent increase in ceftriaxone compared to the same time in 2019. Prescribing then leveled off, though use remained high as the pandemic progressed, according to NHSN data.^27^ Among COVID-19 patients, empiric treatment using broad-spectrum agents was initially common due to concerns for bacterial superinfection,^28^ likely influenced by similar practice patterns for patients admitted with community-acquired pneumonia.^29^ A study performed by Park et al. at OHSU reported high empiric antibiotic use among COVID-19 patients despite low incidence of bacterial coinfection.^30^ Throughout the pandemic, various treatment options, including antibiotics, were explored in an attempt to mitigate COVID-19’s high mortality rate.^28^

The steady decrease in colonization pressure is potentially a key observation that could explain our observed HA-CDI rate. We previously reported that colonization pressure contributes to HA-CDI risk.^31,32^ The decrease in colonization pressure is likely due to a smaller patient population and potentially less patient movement during the pandemic. In a *post hoc* analysis, we observed a steep decline in patient movement (number of physical hospital locations per patient-day) during the first year of the pandemic. There was then a level increase at the start of pandemic year 2, though not back to pre-pandemic levels (Appendix Figure 3). An opposing force to this drop in colonization pressure is longer hospital stays, which could increase an individual’s possibility of either contributing to or experiencing colonization pressure. Unmeasured colonization pressure from colonized patients could also be a factor. Potential changes in *C. difficile* colonization pressure during the COVID-19 pandemic merits further study.

Including an additional interruption point one year into the pandemic to detect potential changes in context improved model fit and suggests a trend increase in HA-CDI rate during the second pandemic period. A possible explanation is that vaccination of healthcare workers and patients could have again altered the healthcare environment. However, because of vaccine availability and the tiered rollout, it is difficult to establish a pre- and post-vaccine period as a specifically defined interruption time point. OHSU began vaccinating all staff, students, and volunteers in January of 2021. As of October 2021, 96 percent of OHSU employees, students, and volunteers were fully vaccinated.^33^ Additionally, in the early pandemic, there were no multiple-occupancy rooms and less contact with healthcare personnel overall. These policies were relaxed in late 2021,^34^ which could explain the apparent uptick in CDI.

One concern for bias is a possible decrease in *C. difficile* testing during the pandemic due to resources and personnel being diverted elsewhere. Although we did find a decrease in the overall volume of *C. difficile* testing, there was an increase in testing on a per encounter basis. We also saw a non-significant decrease in *C. difficile* test positivity (level-change coefficient = -2.8, *P*-value = 0.11) and a significant slope in increase in positivity (slope-change coefficient = 0.27, *P*-value = 0.03) (Appendix Figure 4).

Our study contributes to the growing body of literature around the pandemic’s impact on the healthcare environment. A single-hospital time-series analysis conducted by Aldeyab and colleagues in Ireland reported that a stewardship program aimed at reducing the high-risk antibiotic use successfully reduced use of these agents as well as CDI incidence.^35^ In contrast, our analysis indicated that increases in antibiotic use were not associated with an increase in CDI incidence, providing evidence that other key factors are at play. A retrospective cohort study by Desai et al. examined antibiotic prescribing during the first 11 months of pandemic and observed an initial spike in overall prescribing, which tapered off as the pandemic progressed. The authors suggest that this was driven by prescribing in COVID-19 patients, as guidelines to the contrary had yet to be published.^36^ Nandi and colleagues performed a global cost analysis across 71 countries and highlighted a decrease in sales of broad-spectrum antibiotics in April and May of 2020, followed by a gradual increase to pre-pandemic levels.^37^ Based upon data from NHSN, the US Centers for Disease Control and Prevention (CDC) reported increased overall inpatient prescribing at the onset of the pandemic, but overall lower prescribing in 2021 compared to 2019.^38^ NSHN also reports that outpatient antibiotic prescribing decreased at the onset of the pandemic and then rebounded to pre-pandemic levels.^39^ CDC also reported a decrease in the CDI LabID standardized infection ratio (SIR) across the first 4 quarters of 2020. This is in contrast to consistent increases in the SIR for other healthcare-associated infections, including Methacillin-resistant *Staphylococcus aureus* (MRSA) bacteremia, and catheter, central line, and ventilator-associated events.^40^ We did not observe the same decrease in CDI in our institution.

This study utilized a comprehensive, longitudinal dataset with complete laboratory and pharmacy information that allowed us to apply an accurate case definition for HA-CDI that our group has previously validated.^11^ We also have complete patient location data, which allows us to calculate colonization pressure. Although the interrupted time series design is a strong quasi-experimental study design, the nature of group-level data limits this study’s capacity for causal inference. Our institution is also a low CDI incidence environment compared to the national average (4.2 vs 8.3 cases per 10,000 patient-days per Marra et al.),^14^ which could limit our statistical power and overall generalizability. This could also explain why examining the onset of the delta and omicron COVID-19 variant waves did not yield significant results. Finally, we did not have data on actual infection prevention efforts, so this could not be directly evaluated within our regression model.

While the association between antibiotic use and HA-CDI is well established, it is clear the effect of changes in antibiotic exposure on CDI is sensitive to context, such as shifts in patient mix compared to the pre-pandemic era at many facilities (eg, fewer surgeries, bone marrow transplants, and chemotherapy, all of which was observed at OHSU). Our study raises the hypothesis that COVID-19 prevention measures might have prevented HA-CDI. While the pandemic brought about an increased focus on hand hygiene and global environmental cleaning, our institution’s infection prevention and control team observed no changes in hand hygiene compliance, and no changes were made to the environmental cleaning solutions used. While PPE use was not specifically tracked, there was an increase in protective gown use when available, though shortages were common. Overall, while there was some evidence of an increase in year 2 of the pandemic, more follow-up time across multiple facilities is required to further examine this possible trend.

## Supporting information

Ray et al. supplementary material Ray et al. supplementary material

## Supplementary material

The supplementary material for this article can be found at https://doi.org/10.1017/ice.2024.128
