# Supplementary material for: Examining the impact of the COVID-19 pandemic on hospital-associated Clostridioides difficile infection
Source: Infect Control Hosp Epidemiol. 2024 Oct 21;45(12):1428–34. doi: 10.1017/ice.2024.128 (PMC11663470; doi:10.1017/ice.2024.128)

**Supplementary material**

**Appendix Figure 1.** Time series and trendlines of number of new admissions per time period before and during the pandemic


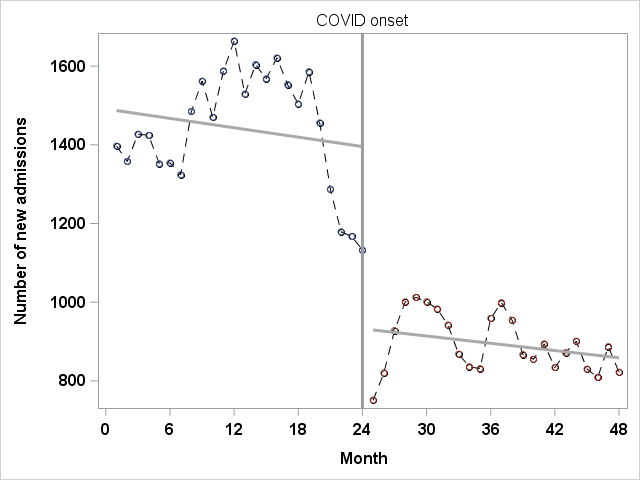


**Appendix Figure 2.** Time series and trendlines of mean number of comorbid conditions per time period before and during the pandemic


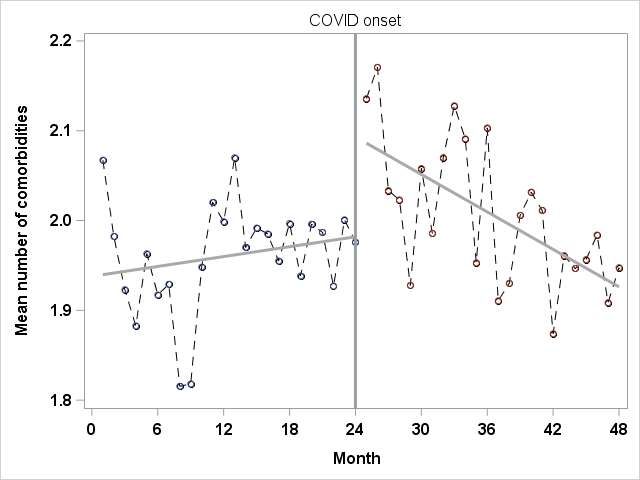


**Appendix Figure 3.** Time series overlay of *C. difficile* test per 100 encounters and *C. difficile* test positivity
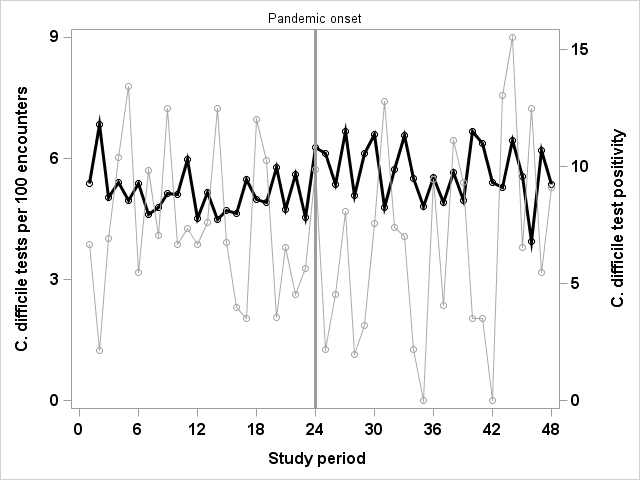


*C. difficile* tests (per 100 encounters)

*C. difficile* test positivity

**Appendix Figure 4.** *Post hoc* time series analysis of number of physical hospital location changes per patient-day.


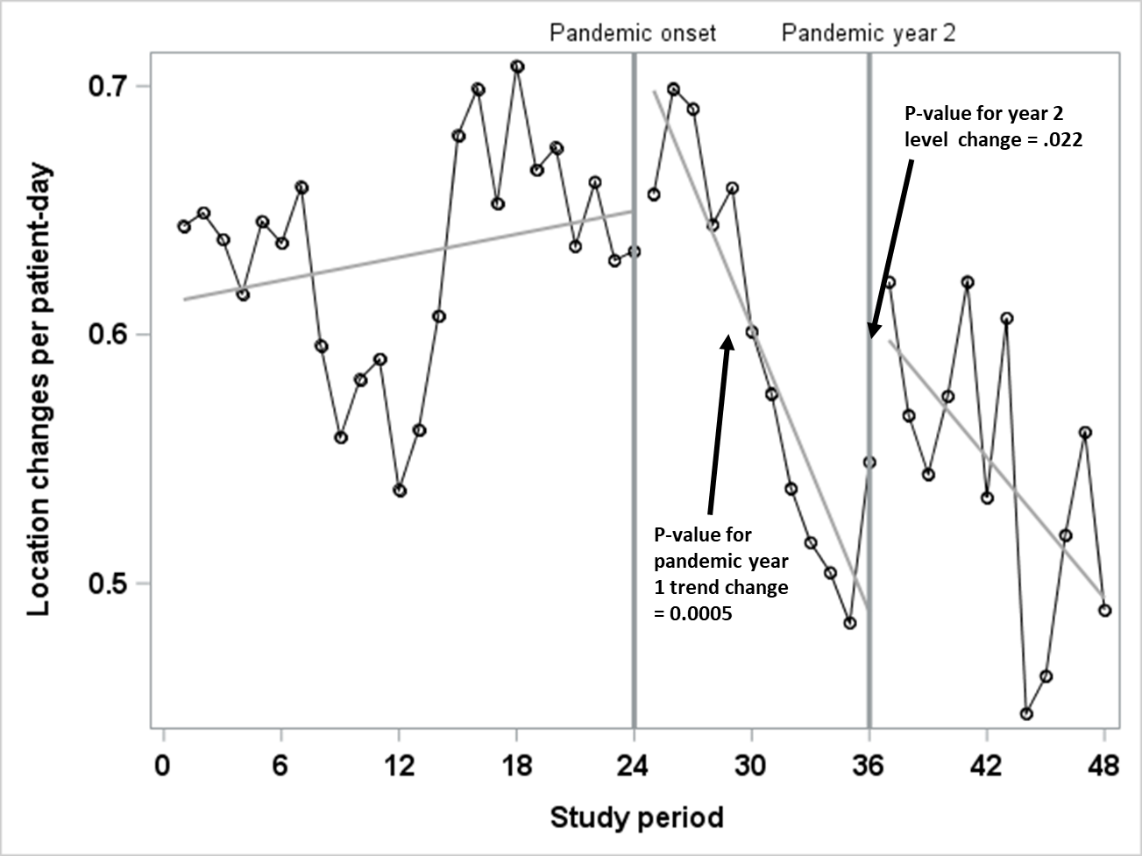

Supplement: Ray et al. supplementary material [file S0899823X24001284sup001.docx]
